# Supplementary material for: A real-time assay for cell-penetrating peptide-mediated delivery of molecular cargos
Source: PLoS One. 2021 Sep 2;16(9):e0254468. doi: 10.1371/journal.pone.0254468 (PMC8412273; doi:10.1371/journal.pone.0254468)
Supplement: S1 Fig — (DOCX) [file pone.0254468.s001.docx]

S1 Fig: Description of plasmids used in the study.

1. pJM140; encodes MBP-CBS in pMAL-c5x

Cloned Sequence:   NdeI and BamHI

CATATGGGTC TGTTCGGCGC GATCGCGGGT TTTATTGAGG GTGGCTGGAC CGGCATGATC

GACGGTTGGT ACGGCAAGCG TCGTTGGAAG AAAAACTTCA TCGCGGTGAG CGCGGCGAAC

CGTTTTAAGA AAATTAGCAG CAGCGGTGCG CTGCCGGGTG CGGCGCACTA CCATCACCAC

CACCACCACT AAGGATCC

Encoded protein (FXa = vector-encoded Factor Xa cleavage site):

*------------------------------Maltose Binding Protein----------------------------------------------------------------------*

MKIEEGKLVIWINGDKGYNGLAEVGKKFEKDTGIKVTVEHPDKLEEKFPQVAATGDGPDIIFWAHDRFGGYAQSGLLAEITPDKAFQDKLYPFTWDAVRYNGKLIAYPIAVEALSLIYNKDLLPNPPKTWEEIPALDKELKAKGKSALMFNLQEPYFTWPLIAADGGYAFKYENGKYDIKDVGVDNAGAKAGLTFLVDLIKN

KHMNADTDYSIAEAAFNKGETAMTINGPWAWSNIDTSKVNYGVTVLPTFKGQPSKPFVGVLSAGINAASPNKELAKEFLENYLLTDEGLEAVNKDKPLGAVALKSYEEELVKDPRIAATMENAQKGEIMPNIPQ-------------------------------------------------------------   ---pMALc5x---------------     *-FXa            --- CBS Tag*

MSAFWYAVRTAVINAASGRQTVDEALKDAQT NSSSNNNNNNNNNNLG  IEGRISHM  KRRWKKN

*-----------------------------------  --Linker--  -His Tag-*

FIAVSAANRFKKISSSGAL PGAAHY HHHHHH STOP

1. pJM161; encodes TAT-NMR-CaM in pET19b

Cloned Sequence:   NdeI and BamHI

catatgcacatgtacggtcgtaagaaacgtcgtcagcgtcgtcgtatgctggtgagcagcaagatcctgcaggttaaaattctgcaaggtaaactgccgctgagcgtggcggcggaaggtgttcagagcgttccgctgtgcctgccgccgcgtgttggtccgagcccgggtccgacccgtagcatcagcgaaaaacacctgctgcgtacccagaacgcgagcggtcagcgtaacccggcggcgccggtggagtggagcctgacccgtcgtgtggtttggtggttcccggcgggcttttggaacaaagcgttcccgccgaaggcgaacgtgattcgtggtcgtggctgccgttgcccgaaaggtggcgaaggtggctgcagcgagcagccgagcgacaccagcttcgttggtgaaatcggcctgtttagcctggcggatcagctgaccgaggaacaaattgcggaatttaaagaggcgttcagcctgtttgacaaggatggtgacggcaccatcaccaccaaggagctgggtaccgtgatgcgtagcctgggtcagaacccgaccgaagcggagctgcaagacatgatcaacgaagttgatgcggacggtaacggcaccattgatttcccggagtttctgaccatgatggcgcgtaagatgaaagataccgacagcgaggaagagatccgtgaggcgttccgtgtttttgataaagacggtaacggctacattagcgcggcggaactgcgtcacgtgatgaccaacctgggcgagaagctgaccgacgaagaggttgatgaaatgatccgtgaggcggatattgacggtgatggccaggtgaactatgaagagttcgttcaaatgatgaccgcgaagtaaggatcc

Encoded protein (*Entk* = vector encodedenterokinase cleavage site):

*-------His Tag---             --Entk--*

MGHHHHHHHHHHSSGHDDDDK HMYGRKKRRQRRRMLVSSKILQVKILQGKLPLSVAAEGVQSVPLCLPPRVGPSP

GPTRSISEKHLLRTQNASGQRNPAAPVEWSLTRRVVWWFPAGFWNKAFPPKANVIRGRGCRCPKGGEGGCSEQPSDTSFVGEIGLFSLADQLTEEQIAEFKEAFSLFDKDGDGTITTKELGTVMRSLGQNPTEAELQDMINEVDADGNGTIDFPEFLTMMARKMKDTDSEEEIREAFRVFDKDGNGYISAAELRHVMTNLGEKLTDEEVDEMIREADIDGDGQVNYEEFVQMMTAK

1. pJM168; MBP-CBS in pMAL-c5x

Cloned Sequence:   NdeI and BamHI

catatgtatggacggaagaaacgaaggcaacgtagacgtccgggtgctgcgcattaccatcatcaccaccaccactaaggatcc

Encoded protein (FXa = vector-encoded Factor Xa cleavage site):

*--------------------Maltose Binding Protein--------------------------------------------------------------------------*

MKIEEGKLVIWINGDKGYNGLAEVGKKFEKDTGIKVTVEHPDKLEEKFPQVAATGDGPDIIFWAHDRFGGYAQSGLLAEITPDKAFQDKLYPFTWDAVRYNGKLIAYPIAVEALSLIYNKDLLPNPPKTWEEIPALDKELKAKGKSALMFNLQEPYFTWPLIAADGGYAFKYENGKYDIKDVGVDNAGAKAGLTFLVDLIKNKHMNADTDYSIAEAAFNKGETAMTINGPWAWSNIDTSKVNYGVTVLPTFKGQPSKPFVGVLSAGINAASPNKELAKEFLENYLLTDEGLEAVNKDKPLGAVALKSYEEELVKDPRIAATMENAQKGEIMPNIPQMSAFWYAVRTAVINAASGRQTVDEALKDAQT

*-------MBP------------------  -Fxa-            ---------TAT---------                   --His-------*

NSSSNNNNNNNNNNLG  IEGR  ISHM YGRKKRRQRRR PGAAHY HHHHHH
