# Supplementary figures and images for: A real-time assay for cell-penetrating peptide-mediated delivery of molecular cargos
Source: PLoS One. 2021 Sep 2;16(9):e0254468. doi: 10.1371/journal.pone.0254468 (PMC8412273; doi:10.1371/journal.pone.0254468)

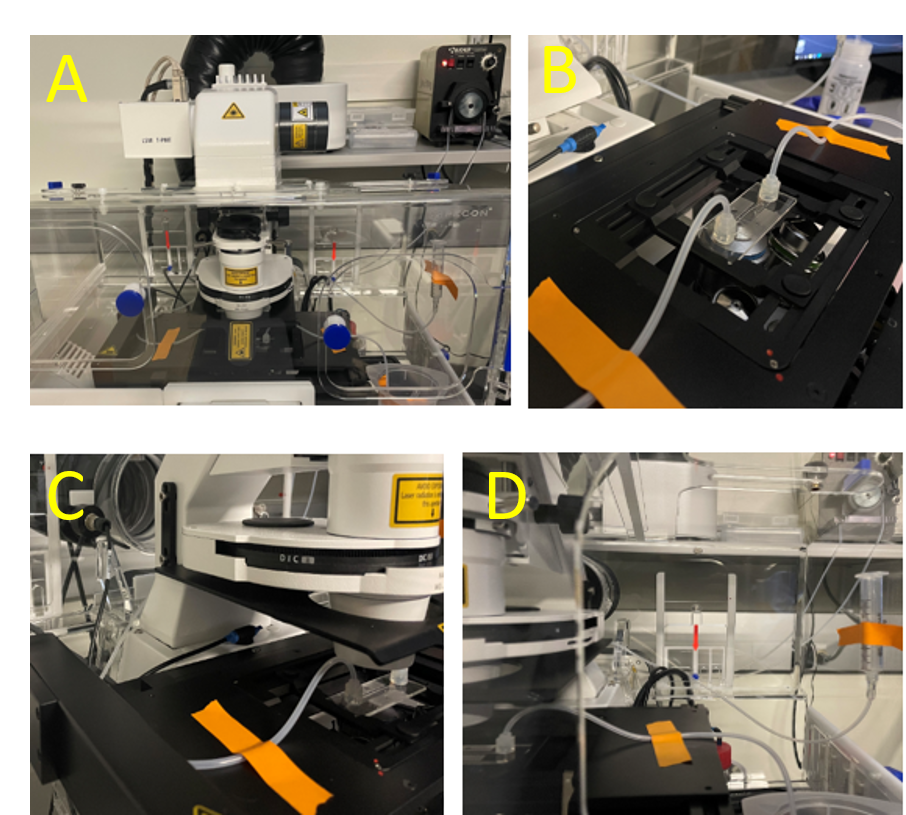

Supplement: S2 Fig — A, overview of tubing system set up from outside of incubator case. B, channel slide attached to tubing system, sitting above 40X objective. C, left side view of channel slide and tubing set up. Tubing is inserted through the back of the incubator and run around the back side of the microscope. D, right side view of channel slide and tubing set up. Tubing runs from reservoir, through the back of the incubator case, and up to the peristaltic pump. Tubing runs through the pump and comes back through the back side of the incubator where it is run around the back of the microscope to the left side of channel. Right side of channel runs via tubing to waste. (TIF) [file pone.0254468.s002.tif]

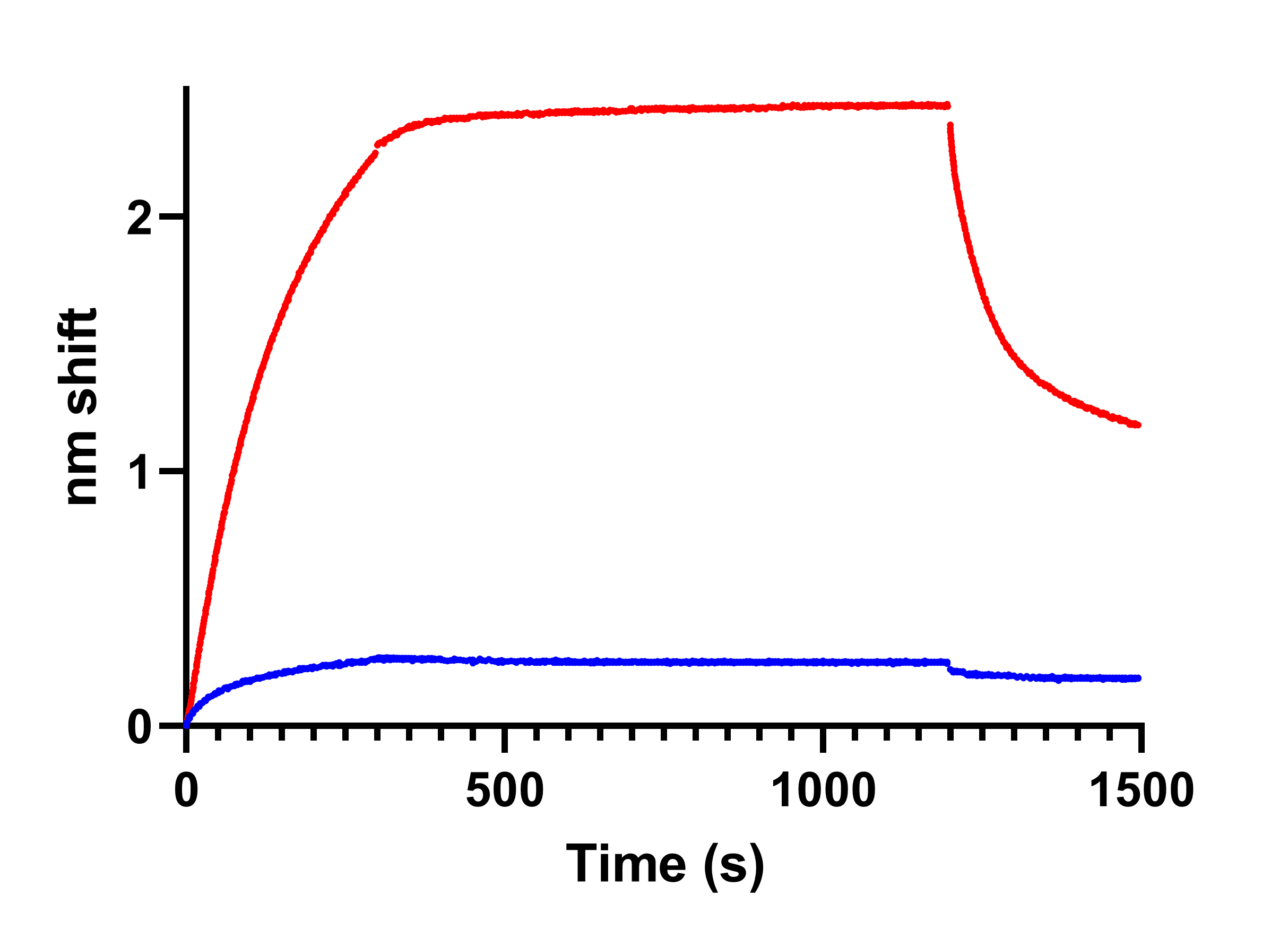

Supplement: S3 Fig — BLI experiment in which 1 μM TAT-NMR-CaM was exposed as analyte to a sensor with biotinylated CBS-MBP (red) or a sensor without any tethered ligand (blue). (TIF) [file pone.0254468.s003.tif]
