# Supplementary material for: A real-time assay for cell-penetrating peptide-mediated delivery of molecular cargos
Source: PLoS One. 2021 Sep 2;16(9):e0254468. doi: 10.1371/journal.pone.0254468 (PMC8412273; doi:10.1371/journal.pone.0254468)
Supplement: Attachment — Submitted filename: Response to Reviewers.docx [file pone.0254468.s008.docx]

August 10, 2021

Dr. Eugene A. Permyakov

Academic Editor

PLOS ONE

RE: Manuscript PONE-D-21-20501

Dear Dr. Permyakov,

Thank you for considering our manuscript, PONE-D-21-20501, for publication in PLOS ONE. We are appreciative of your and the reviewers’ efforts and honored that they found merit in our study and made constructive criticisms of the manuscript. We have made the suggested revisions, described in full below, and are proud to resubmit an improved, revised manuscript for further consideration.

Our answers to editorial and reviewer points:

Editor:

1) We have done our best to ensure that our manuscript meets the style requirements.
2) Competing Interests/Financial Disclosure. We are afraid there was a miscommunication in the disclosure. While two authors do have equity interest in New Echota Biotechnology, a start-up biotechnology company that has license to a patent on the CPP-adaptor technology, the study was not funded by any commercial source at all. Thus your statement that “We note you received funding from a commercial source: JLM and SJN” is in error. JLM and SJN are the initials of the authors, Jonathan L. McMurry and Scott J. Nowak, who have equity in the company. Both are employed by Kennesaw State University and were not supported or paid by the company in any way. The company has no funding and is mostly a dormant entity at the moment. We have amended the Competing Interests statement to eliminate the use of initials to avoid the miscommunication:

I have read the journal's policy and the authors of this manuscript have the following competing interests: Authors McMurry and Nowak have equity interest in New Echota Biotechnology, a start-up company which has exclusive license to patent US10654894B2 on the CPP-adaptor technology and applications thereof. The other authors have no competing interests. This does not alter our adherence to PLOS ONE policies on sharing data and materials.

Please let us know if you need additional changes.

3) We have added captions for the Supporting Information files as requested.
4) We have eliminated the mention of data not shown, in one case providing a new figure in Supplemental Information and in the other referring to the paper with the data that was not shown.

Reviewer #1:

1) Reviewer #1 notes in “Major Comments” that the superiority of the real-time assay to the static endpoint one is insufficiently presented. We have added a summary paragraph that more clearly describes the advantages of our method, lines 380-396.
2) The reviewer points out that the penetration mechanisms remain unclear (indeed they are a matter of great debate in the field) and implies that we should repeat the experiments “focusing on the key factors,” which include ”cytoskeleton, tubulin, pH change” and others. We completely agree that these and other factors are necessary to understanding the mechanisms and kinetics of CPP-mediated entry and delivery of cargo, but ask why is it we, of all the thousands of CPP studies are to be held to knowing the mechanisms that have for decades eluded understanding? We would instead plead that the study warrants publication now as it stand precisely so that other researchers with the resources to conduct those studies might incorporate our or similar assays in their work to better understand exactly these factors. We note that we did focus on some of the factors in our small molecule inhibitors experiments shown in Fig. 8 and the negative results we obtained strongly suggest that the mechanistic answers are likely very complex and will yet be a long time coming. We will continue to pursue them, but the scope of what the reviewer implies need be done is well beyond our ability to do at present and will require the efforts of the entire field. Best that they know about our assay now, we think. We hope the reviewer agrees.
3) Reviewer asks if other types of proteins can be excreted from the endosome. The brief answer is yes. Endosomal escape is recognized as the largest technical hurdle to be overcome in the development of CPP-based therapeutics. We direct him or her to our prior papers describing our system (references # 10 and 11, and our review on the subject, #6). Salerno et al. demonstrates delivery of three cargo proteins that vary in size and oligomerization. In Ngwa, et al., we delivered a model cargo protein to the nucleus, endoplasmic reticulum and lysosomes using appropriate signal peptides. The reviewer is absolutely correct that it is unclear how proteins escape the endosome, but we have shown that they do (i.e. how else would they wind up in the nucleus?). The distance analysis presented in the present study represents an advance in that we can now, at any moment in time during the course of a real-time assay, quantify how much better our CPP-adaptors deliver cargo than the standard linkage; it can be a tool to measure endosomal escape going forward. We have modified comments in the last paragraph to address this critique.
4) Figure mistakes: we have corrected them according to the reviewer’s comments. We also apologize as these are the sorts of errors we endeavor never to make, but somehow usually do. Thanks to the reviewer for catching them and sorry to have made doing so necessary.

Reviewer #2: Reviewer #2 had no concerns that needed addressing in this letter.

We also made a very few other minor edits where we caught typos or realized we could enhance clarity. We also neglected to put an umlaut in Göettingen in the materials and methods sections. The changes are marked on the track changes document.

Again, we thank you and the reviewers for your efforts. They have resulted in a substantially improved manuscript we hope you will find worthy of publication.

Sincerely,

Jonathan McMurry, Ph.D.

Corresponding Author
